# Mechanism Analysis and Potential Applications of Atomic Oxygen Erosion Protection for Kapton-Type Polyimide Based on Molecular Dynamics Simulations

**DOI:** 10.3390/polym16121687

**Published:** 2024-06-13

**Authors:** Shengrui Zhou, Li Zhang, Liang Zou, Bilal Iqbal Ayubi, Yiwei Wang

**Affiliations:** School of Electrical Engineering, Shandong University, Jinan 250061, China

**Keywords:** AO, ReaxFF MD, PI, FPI, POSS, erosion

## Abstract

Polyimide (PI) is widely used in aerospace applications due to its excellent properties. However, the high concentration of atomic oxygen (AO) in low-earth orbit (LEO) significantly degrades its performance. This study employs reactive molecular dynamics (MD) simulations to analyze the AO erosion resistance of fluorinated polyimide (FPI) and polyhedral oligomeric silsesquioxane (POSS) composite polyimide models. The 35 ps simulation results indicate that the PI/POSS composite exhibits the best protective performance. The protection mechanism involves the formation of an SiO_2_ carbonized layer that prevents the transmission of AO and heat to the polyimide matrix, resulting in a normalized mass of 84.1% after erosion. The FPI model shows the second-best protective effect, where the introduction of -CF_3_ groups enhances the thermal stability of the polyimide matrix, resulting in a normalized mass of 80.7% after erosion. This study explores the protective effects and mechanisms of different polyimide protection methods at the molecular level, providing new insights for the design of AO erosion protection systems.

## 1. Introduction

Polyimide (PI) is widely used in the aerospace sector due to its outstanding tolerance to extreme temperatures, electrical insulation capabilities, and mechanical strength [1,2,3,4]. However, in low-earth orbit (LEO), spacecraft are exposed to various threats such as atomic oxygen (AO), severe ultraviolet radiation, X-rays, and rapid temperature changes [5]. Among these, atomic oxygen is a significant hazard to spacecraft polymer materials because of its high reactive activity and substantial presence in LEO. The relative impact energy of atomic oxygen on spacecraft, flying at the first cosmic velocity in LEO, is between 4.5 and 5 eV (equivalent to a relative speed of 7 to 8 km/s) [6,7], leading to varying levels of insulation material erosion and significantly reducing spacecraft operational lifespan [8,9,10,11]. Therefore, exploring the methodologies for polyimide’s resistance against atomic oxygen erosion is of paramount importance.

Presently, the research on mitigating the erosion of spacecraft insulating materials by atomic oxygen has concentrated on two main strategies. The first involves applying enhanced protective coatings on the polymer surface, with typical layers including graphene [12,13,14], SiO_2_ [15], and polyhedral oligomeric silsesquioxane [16,17,18,19,20,21,22]. Notably, the study of POSS-enhanced polymer resistance against atomic oxygen has drawn significant interest due to POSS’s ceramicization capability, which under specific conditions can transform into a highly dense, transparent SiO_x_ layer exhibiting excellent thermal insulation, flame retardance, and atomic oxygen erosion resistance [23,24,25]. Xu and colleagues achieved exceptional anti-AO erosion capabilities with SiO_2_/POSS/Kapton samples prepared via radio-frequency magnetron sputtering technique, showing varying growth times [26]. Timothy and co-researchers demonstrated that polyimide films containing POSS and coated with an atomic layer deposition Al_2_O_3_ layer effectively reduced the energy transfer from AO, significantly decreasing Kapton’s atomic oxygen erosion rate [27]. However, once the protective coating cracks or even peels off, the PI will be exposed again to the AO environment and undergo degradation [28].

Another strategy is by altering PI’s chemical structure itself through the incorporation of elements such as fluorine, phosphorus, or silicon [29,30]. These elements form a passivation layer under continuous AO erosion, effectively diminishing the erosion impact of AO on polyimide [31,32]. The study of Chen et al. on transparent polyimide films containing side-chain diphenyl phosphine oxide and trifluoromethyl groups showed that the modified polyimide’s erosion rate was only 18.7% of Kapton’s film [29]. Zhang and others explored the degradation behavior of a fluorine-containing colorless polyimide under an AO environment through ground exposure experiments, finding that the modified polyimide films exhibited lower erosion rates and better AO tolerance [30]. Wang and co-authors prepared polyimide films based on multi-ring fluorinated dianhydride and phosphorus-containing diamine, discovering that the modified polyimide films displayed higher glass transition temperatures, lower linear thermal expansion coefficients, and outstanding atomic oxygen resistance [33]. These results suggest that the introduction of fluorine and phosphorus elements significantly enhances the thermal stability and AO erosion resistance of polyimides. However, introducing other elements into the main chain of PI can alter the original properties of PI, and the preparation process is complex and costly [34].

Conducting actual space and ground exposure experiments is an effective method to study the polymer cracking mechanisms under the influence of AO erosion [35,36]. However, the mechanisms behind the passivation layer’s formation and the pathways for the generation of atomic oxygen erosion products remain unclear. Thus, understanding the degradation mechanisms of polymers under AO erosion and the intermediate reaction processes at the molecular level becomes especially important.

Up until now, studies have employed distributed computing to perform comprehensive atom-level molecular dynamics (MDs) simulations, aiming to evaluate the decomposition patterns of matrix materials in harsh environments. Rahmani and their group utilized reactive molecular dynamics simulation techniques to investigate the effects of embedding nanoparticles of various kinds and orientations in polyimide for enhancing its resistance to atomic oxygen bombardment [37]. Zeng and co-researchers utilized a ReaxFF MD simulation to investigate the decomposition behaviors of polyvinylidene fluoride, fluorinated polyphenylsilsesquioxane, and their composites under atomic oxygen impact, finding that incorporating FP-POSS into PVDF effectively bolstered its AO erosion stability [38].

Given these considerations, this study thoroughly investigates the resistance of fluorinated polyimide (FPI) and POSS/PI nanocomposite materials against AO bombardment through ReaxFF molecular dynamics. We quantitatively assess the temperature changes, product composition, mass depletion, and damage propagation depth in the polymer system under atomic oxygen impact, revealing its degradation and reaction mechanisms. This work provides a comparative analysis of the AO erosion resistance of fluorine-containing polyimide and POSS-blended polyimide materials, offering theoretical guidance for the structural and integrated design of AO-resistant molecular materials.

## 2. Methods and Models

This study employed the Reax package in LAMMPS for simulating atomic oxygen collisions, using the ReaxFF force field to continuously update and analyze bond lengths, bond orders, and the correlation between bond orders and bond energies throughout each step of the molecular dynamic simulations [39,40], allowing for the continuous formation and breaking of bonds during the simulation process. ReaxFF segments the system’s energy into a multitude of specific energy contributions, which are bifurcated into two primary categories: valence terms and nonbonded terms. The valence terms encompass bond energy (*E_bond_*), wherein the bond order is factored in; over/under-coordination energy (*E_over_* and *E_under_*), serving as penalty/remediation mechanisms for addressing the atoms’ over-coordination or under-coordination within a molecule; valence angle energy (*E_val_*); penalty energy applicable to double bonds that share an atom within a valence angle (*E_pen_*); torsional angle energy (*E_tor_*); and conjugated energy (*E_conj_*), among others. The nonbonded terms are comprised of van der Waals energy (*E_vdWaals_*) and Coulombic energy (*E_Coulomb_*). In this research, a specific set of ReaxFF force field parameters formulated by van Duin et al. [41] was utilized, noted for their proven accuracy in simulating the dynamic processes of atomic oxygen erosion on polyimide in space.

**Figure 1:**
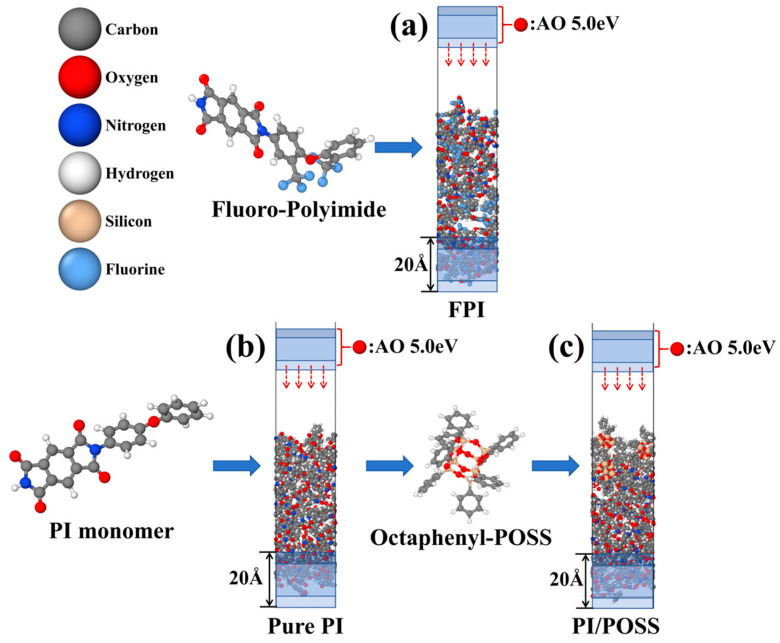
(**a**) Fluoro-polyimide model; (**b**) pure PI model; (**c**) PI/POSS nanocomposites model.

Figure 1 displays the molecular configurations and three types of equilibrium cell structures used in this study. Initially, a simulation box populated with 26 Kapton-PI monomers and dimensions of 20 Å × 20 Å × 40 Å was prepared for the model. This model underwent relaxation under NPT conditions (constant pressure and temperature) aimed at 298 K and atmospheric pressure to form a dense cellular structure. Throughout this phase, the cell density diminished to a stable equilibrium density of 1.37 g/cm^3^, aligning closely with the empirical density of 1.41 ± 0.2 g/cm^3^, and achieving final dimensions of 20.02 Å × 20.02 Å × 30.04 Å. Additionally, a 20 Å substrate buffer layer was affixed to the model’s base and secured, while the vertical periodic boundary was eliminated, extending the model to 120 Å. Finally, the model underwent relaxation and equilibrium under NVT (constant volume, constant temperature) ensemble conditions at 300 K for 500 ps.

The same process was applied to model the fluoro-polyimide filled with 22 units and the PI/POSS nanocomposite material with a mass fraction of 12.88% for polyhedral oligomeric silsesquioxane cages, yielding equilibrium densities of ρ_FPI_ = 1.49 g/cm^3^ and ρ_PI/POSS_ = 1.33 g/cm^3^, respectively. The stable cell dimensions were 19.28 Å × 19.28 Å × 28.20 Å for FPI and 20.97 Å × 20.97 Å × 34.69 Å for PI/POSS. The PI/POSS nanocomposite material consists of 22 PI monomers and three branch-connected octaphenyl silsesquioxanes. In this study, the modeling process was carried out using BIOVIA Materials Studio (2020).

In the LEO environment, the density of AO is approximately 10^7^ to 10^10^ atoms/cm^3^, with a kinetic energy of around 4.5 to 5 eV, depending on the altitude of the orbit [42,43,44]. In this computational analysis, AO with a kinetic energy of 5 eV is introduced 50 Å above the substrate’s surface at a rate of 5 atoms per picosecond, and the simulation employs a time step of 0.1 femtoseconds. To monitor the structural alterations within the system caused by AO erosion visually, this study utilized the NVE ensemble for simulating AO erosion across three substrate models, ensuring all kinetic energy from AO was transformed into modifications in the system’s surface structure.

## 3. Results and Discussion

### 3.1. Erosion Kinetics of Pure PI, FPI, and PI/POSS Nanocomposite Materials

**Figure 2:**
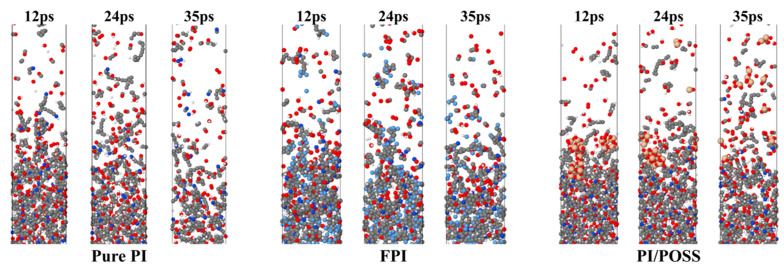
Snapshots of PI, FPI, and PI/POSS systems during the AO erosion process.

Figure 2 depicts the alterations in structure experienced by three models throughout the AO erosion phase. It is evident from the figure that all three systems suffer severe damage after AO impact, with molecules beginning to dissociate into numerous gaseous molecular fragments. The polymer structure at the bottom of the pure PI exhibits signs of loosening and fragmentation, while the bottom polymers of the FPI and PI/POSS systems remain relatively dense and intact. It is also observed that the overall damage to the FPI and PI/POSS models is less than that to the pure PI model. In the process of AO erosion, the PI/POSS model produces a significant volume of SiO_x_ byproducts, which act as a barrier against the energy transfer from atomic oxygen, thereby markedly diminishing the effects of AO on the polymer.

**Figure 3:**
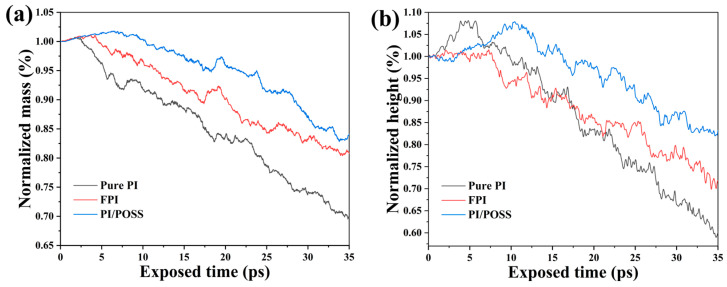
Changes in (**a**) normalized mass and (**b**) normalized height during AO erosion.

To measure the erosion resistance offered by the three models, both the normalized mass and the normalized height of the polymer substrates were assessed during the erosion. The normalized mass is calculated by dividing the mass left behind post-AO erosion by the original mass of the substrate. Similarly, the normalized height is determined by dividing the substrate’s average height post-erosion by its original height. These metrics, illustrated in Figure 3, provide insight into the durability of the substrates against AO erosion; the normalized mass and height of the pure PI model decrease the fastest, indicating that the PI/POSS has the best protective effect, with FPI’s protection effect being intermediate. During the initial stages of AO erosion, the normalized mass of all three systems shows varying degrees of increase, suggesting that a small amount of energy injected by AO has not reached the threshold for significant deterioration of polymer molecules, displaying varying degrees of resistance to AO erosion across the models.

**Figure 4:**
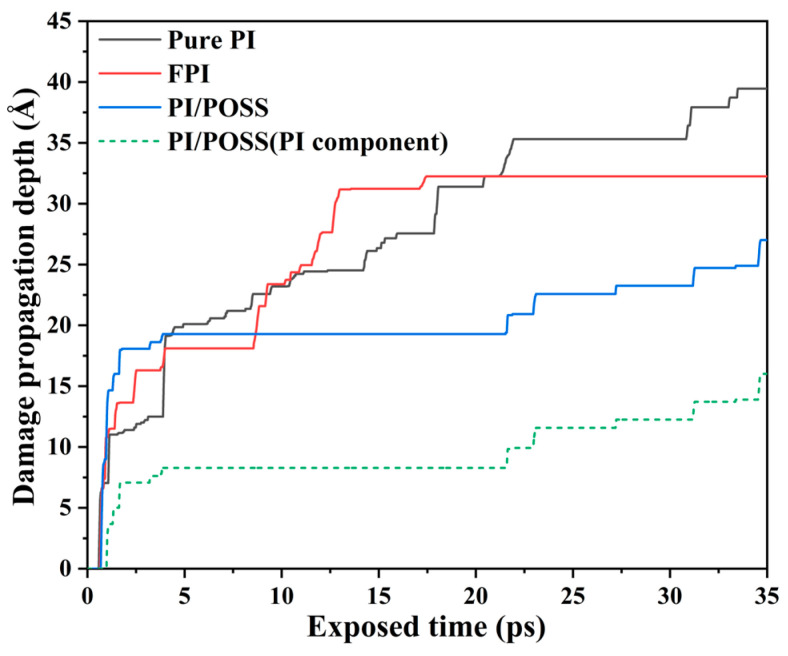
AO erosion process in model’s damage propagation depth.

The extraction of the maximum depth of damage propagation during the AO erosion process revealed that the green dashed line, as shown in Figure 4, represents the depth of damage propagation in the PI component beneath the POSS layer in the PI/POSS system. It is observable that compared to the pure PI system, the PI/POSS exhibits the most effective protection, with the POSS coating substantially absorbing and preventing AO from penetrating into the PI matrix. Relative to the other two configurations, the depth of AO erosion damage propagation in the FPI reaches its peak first and remains stable. This stability arises because the continuous erosion by AO amplifies the thermal motion within the polymer matrix, leading to the initiation of dissociation and sparse distribution of internal PI molecules, thereby facilitating the penetration of active AO through the polymer matrix. However, fluorinated PI maintains a better structural stability at the same temperature, thereby reducing the spread of AO damage to a certain extent.

### 3.2. Temperature Evolution during AO Erosion

**Figure 5:**
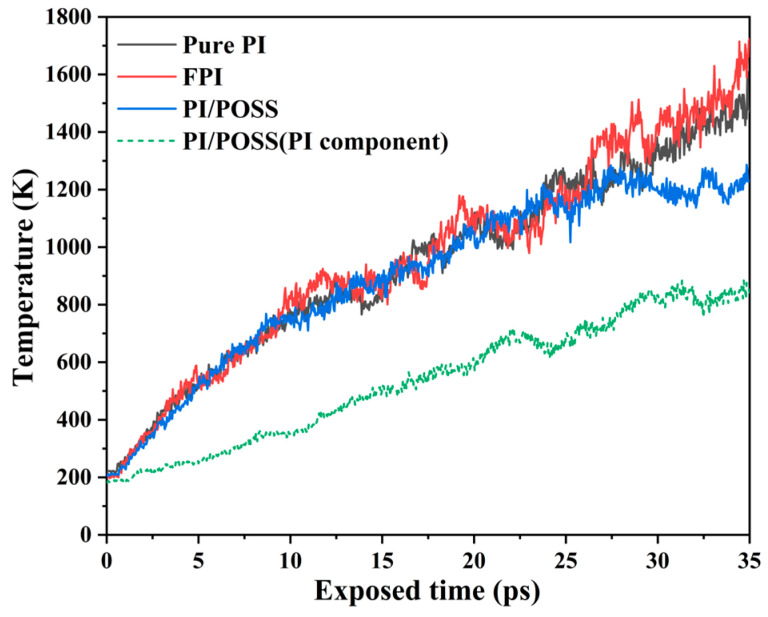
Temperature evolution curves of three PI models.

To study the temperature evolution patterns of the three models under AO impact, their temperature distributions were calculated, as shown in Figure 5. The temperature change trend in the pure PI system was almost identical to that of the FPI, with the temperature of the FPI system slightly higher than that of the pure PI system after 35 ps of AO erosion, both reaching around 1600 K. Near 27 ps, the temperature of the PI/POSS system peaked due to, on one hand, the extensive AO erosion converting POSS into SiO_X_ covering the PI surface, effectively preventing AO from eroding the PI matrix beneath the POSS. On the other hand, at this point, the temperature had reached the system’s decomposition threshold, causing polymer molecules to break down into numerous gaseous small molecular fragments. These fragments, floating above the polymer, not only prevented AO from colliding with PI but also carried away a significant amount of heat from the system, stabilizing the temperature around the 1200 K threshold, and even showing a tendency for the temperature to decrease. The presence of POSS in the PI/POSS (PI component) effectively prevented heat from transferring downward. In contrast, for the pure PI and FPI systems, without a physical protective layer, the majority of the AO impacts were converted into temperature increases in the system. It is evident that the reduction in mass and height of the pure PI and FPI systems is due to thermal decomposition caused by AO’s physical impact and the rise in temperature. Moreover, it can be inferred that the introduction of trifluoromethyl groups gives FPI a higher decomposition temperature compared to pure PI.

**Figure 6:**
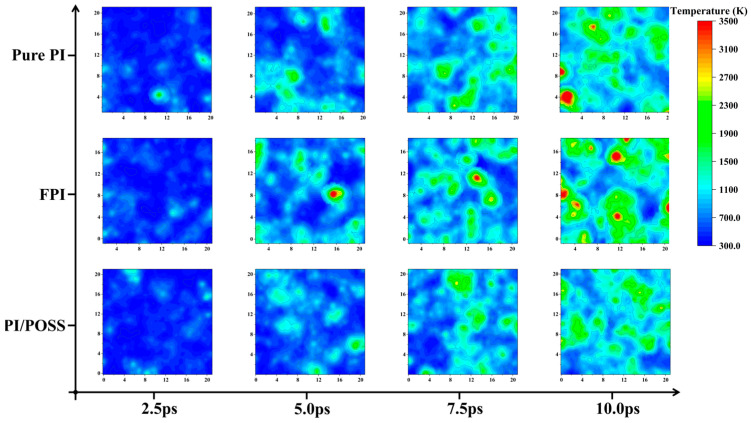
Temperature distribution at Z = 40 Å for three models at different times.

Further, the local temperatures at Z = 40 Å of the three models were calculated at 2.5 ps, 5.0 ps, 7.5 ps, and 10.0 ps during AO erosion, as depicted in Figure 6. In the initial stages of AO erosion (first 5 ps), the temperature distributions of the three models were similar; from 7.5 ps onwards, compared to the PI/POSS system, local hotspots appeared in the pure PI and FPI systems, indicating that at this moment, AO had penetrated the upper polymer and transferred heat to that area. The temperature rise in the PI/POSS was mainly caused by heat transfer from above. The SiO_2_ ceramic carbon layer hardly reacted with AO, and the free radicals and SiO_x_ together hindered AO erosion. For the PI/POSS model, no significant local hotspots were observed, resulting in a more uniform temperature distribution.

### 3.3. Analysis of AO Erosion Byproducts

**Figure 7:**
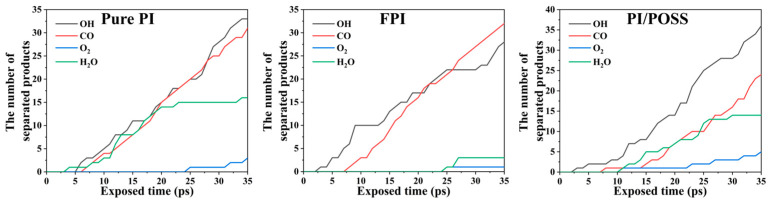
Statistics on the small molecule products of pure PI, FPI, and PI/POSS.

During the simulation, the types and quantities of small molecular substances separated from the substrate surface by more than 10 Å were recorded every 1 ps, as shown in Figure 7. It was found that under the influence of AO, the production of OH and CO in all three systems exceeded that of O_2_ and H_2_O, with the contents of the former two nearly identical in both the pure PI and FPI systems. This occurs as hydroxyl free radicals and CO molecules form upon the interaction of atomic oxygen with both intact and degraded polymer molecules, where PI molecules and the phenyl rings in POSS act as sources for these free radicals. A notable feature of FPI is the production of less H_2_O, as the introduction of trifluoromethyl groups affects the molecular arrangement and structural density of the polymer chain. The strong electronegativity of trifluoromethyl inhibits the supply of free H radicals, making it difficult for hydroxyl to combine with free H atoms to form H_2_O molecules. It can be deduced that FPI’s better AO erosion resistance compared to pure PI is dominated by its molecular chemical stability, with POSS serving more as a physical barrier to AO erosion.

**Figure 8:**
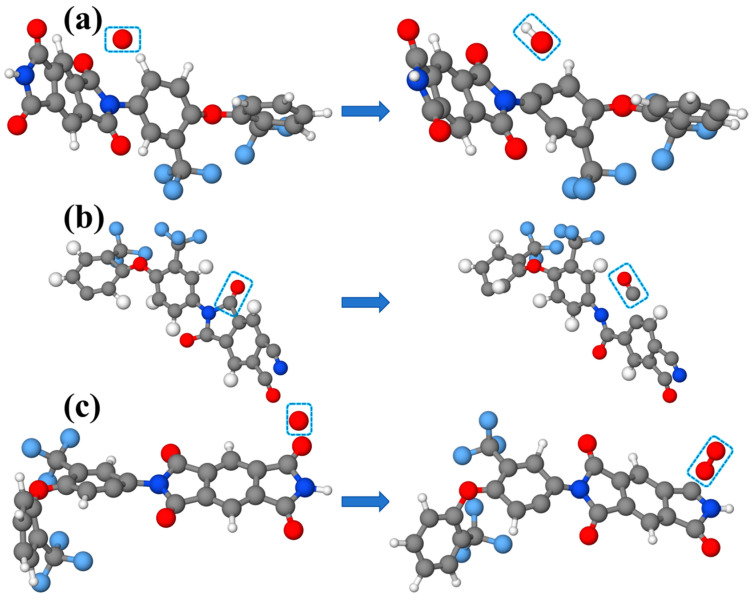
Formation processes of (**a**) OH, (**b**) CO, and (**c**) O_2_ in the FPI model.

Taking the FPI system as an example, reaction snapshots of OH, CO, and O_2_ were extracted, as illustrated in Figure 8. OH is primarily generated by the dehydrogenation reaction with AO. With continuous AO erosion, the system’s temperature rises, and when it reaches the decomposition temperature of FPI, the C-N bonds break, releasing CO molecules. AO collision with the imide ring generates O_2_ molecules, showing that the introduction of highly polar -CF_3_ improves the overall molecular stability, making the -CF_3_ group less likely to react with AO and overall slowing down the erosion process by AO.

## 4. Conclusions

This study constructed FPI and PI/POSS models through molecular dynamics simulations and conducted AO erosion simulations to investigate the degradation process of polyimide under AO erosion in low-earth orbit. This study revealed the mechanisms and effects of various protective measures for PI under AO impact. The key findings include the following:

(1) The simulation of AO erosion on pure PI, FPI, and PI/POSS showed that AO erosion of polymers manifests in the two following aspects: thermal decomposition and oxidative erosion caused by the temperature rise induced by AO. PI/POSS exhibited the best protective effect, with POSS converting into a significant amount of SiO_X_ under AO erosion, preventing further AO penetration into the PI matrix. FPI’s protective effect was second, with the introduction of -CF_3_ groups enhancing the polymer’s stability and thus showing better AO resistance.
(2) The temperature rise in FPI was similar to that in the pure PI system. However, adding POSS and converting it into SiO_X_ products under AO erosion effectively prevented heat transfer to the PI matrix. The temperature rise of the polyimide molecules beneath the POSS coating was effectively suppressed, preventing extensive thermal decomposition of the polyimide molecules and thus reducing the overall mass loss.
(3) Under AO erosion, all three systems produced a significant amount of gaseous small molecular products, mainly CO and OH, related to the abundant carbon chains in polyimide and phenyl groups in POSS.

This study provides a comparative analysis of the AO erosion protective effects of pure PI, FPI, and PI/POSS at the atomic scale, extracting the temperature evolution process during AO erosion, and revealing the reaction mechanisms of AO erosion and the formation mechanism of small molecular products. This work offers a new perspective for the study of polyimide polymer films’ resistance to AO erosion and provides theoretical support for related research in the field of spacecraft resistance to AO erosion in low-earth orbit.

## Data Availability

The data that support the findings of this study are available upon reasonable request from the authors.

## References

[1] Xi S., Wang Y., Zhang X., Cao K., Su J., Shen J., Wang X. (2023). Fire-resistant polyimide-silica aerogel composite aerogels with low shrinkage, low density, and high hydrophobicity for aerospace applications. Polym. Test..

[2] Li Q., Guo Y., Ouyang C., Yi S., Liu S. (2023). Porous highly fluorinated polyimide/polydopamine nanocomposite films with simultaneously enhanced toughness, UV-shielding, and photostability for aerospace applications. Polym. Test..

[3] Jia T., Chen H., Fan Z., Xu H., Huang J., Wang P., Xing H., Jia H., Fan X., Zhou H. (2023). High-strength and high-temperature-resistant multilayer interconnected polyimide paper derived from anisotropic aerogel via a hot-extrusion strategy for aerospace applications. Appl. Surf. Sci..

[4] Luo H., Liu J., Yang Z., Zhang Q., Ao H., Wan Y. (2020). Manipulating thermal conductivity of polyimide composites by hybridizing micro- and nano-sized aluminum nitride for potential aerospace usage. J. Thermoplast. Compos. Mater..

[5] Srinivasan S.G., van Duin A.C.T. (2011). Molecular-dynamics-based study of the collisions of hyperthermal atomic oxygen with graphene using the ReaxFF reactive force field. J. Phys. Chem. A..

[6] Li G., Wang J., Niu B., Xing Y., Liang X., Zhang Y., Long D. (2023). Atomic Oxygen-Induced Surface Erosion Behavior and Mechanical Degradation of Polyether Ether Ketone via Reactive Molecular Dynamics Simulations. J. Phys. Chem. B.

[7] Chowdhury A., Vashisth A., Bakis C.E., van Duin A.C.T. (2019). Reactive Molecular Dynamics Simulations of the Atomic Oxygen Impact on Epoxies with Different Chemistries. J. Phys. Chem. C.

[8] Suliga A., Jakubczyk M.E., Hamerton I., Viquerat A. (2018). Analysis of atomic oxygen and ultraviolet exposure effects on cycloaliphatic epoxy resins reinforced with octa-functional POSS. Acta Astronaut..

[9] Clausi M., Santonicola G.M., Schirone L., Laurenzi S. (2017). Analysis of ultraviolet exposure effects on the surface properties of epoxy/graphene nanocomposite films on Mylar substrate. Acta Astronaut..

[10] Andropova U.S., Chernik V.N., Novikov L.S., Sapozhnikov D.A., Tebeneva N.A., Aysin R.R., Serenko O.A. (2024). Effect of nanoparticles and siloxane groups on the atomic oxygen erosion resistance of copolyimides. Polym. Degrad. Stab..

[11] Chen L., Li Z., Lee C., Wang X., Zhang Y., Liu M., Brown T., Smith J., Patel R., Kim H. (2016). Unified model for low-Earth-orbital atomic-oxygen and atomic-oxygen/ultraviolet induced erosion of polymeric materials. Aerosp. Sci. Technol..

[12] Yizhi Z., Zhigang S., Xiaojing Z. (2021). Exploring graphene and graphene/nanoparticles as fillers to enhance atomic oxygen corrosion resistance of polyimide films. Colloids Surf. A..

[13] Liu L., Shen Z., Liang S., Yi M., Zhang X., Ma S., Wang P., Zhou Q., Lin J., Gao R. (2013). Enhanced atomic oxygen erosion resistance and mechanical properties of graphene/cellulose acetate composite films. J. Appl. Polym..

[14] Young J.N., Bo S.J., Gon C.K. (2015). Characteristics of Silane Treated Graphene Filled Nanocomposites Exposed to Low Earth Orbit Space Environment. Compos. Res..

[15] Wang D.S., Mi S.M., Liu T.Z., Li M.S. (2011). SiO_2_ Coatings Prepared by Sol-Gel Process Protecting Silver from Atomic Oxygen Erosion. Appl. Mech. Mater..

[16] Minton T.K., Wright M.E., Tomczak S.J., Marquez S.A., Shen L., Brunsvold A.L., Petteys B.J., Doe J., Roe J., Smith A. (2012). Atomic oxygen effects on POSS polyimides in low earth orbit. ACS. Appl. Mater. Interfaces.

[17] Yu Q., Wang X., Zhang C., Yang Z., Cheng G., Yang Z., Liu W., Smith A., Johnson B., Davis C. (2023). Resistance to space atomic oxygen radiation of MAC-based supramolecular gel lubricant containing POSS. Tribol. Int..

[18] Duo S., Chang Y., Liu T., Zhang H. (2013). Atomic Oxygen Erosion Resistance of Polysiloxane/POSS Hybrid Coatings on Kapton. Phys. Procedia.

[19] Li W., Liu H., Feng L. (2013). Preparation and anti-atomic oxygen erosion properties of OPPOSS/PI composites. Int. J. Miner. Metall. Mater..

[20] Liu Y., Sun Y., Zeng F., Wang G., Zhao H., Liu M., Chen X., Li J., Xu K., Huang L. (2014). Characterization and analysis on atomic oxygen resistance of POSS/PVDF composites. Appl. Surf. Sci..

[21] Fang G., Li H., Liu J., Ni H., Yang H., Yang S. (2015). Intrinsically Atomic-oxygen-resistant POSS-containing Polyimide Aerogels: Synthesis and Characterization. Chem. Lett..

[22] Lei X., Qiao M., Tian L., Chen Y., Zhang Q., Wang L., Zhao P., Li Z., Zhang R., Sun J. (2015). Evolution of surface chemistry and morphology of hyperbranched polysiloxane polyimides in simulated atomic oxygen environment. Corros. Sci..

[23] Yuan G., Yang B., Chen Y., Jia Y. (2019). Synthesis of a novel multi-structure synergistic POSS-GO-DOPO ternary graft flame retardant and its application in polypropylene. Compos. Part A.

[24] Atar N., Grossman E., Gouzman I., Bolker A., Murray V.J., Marshall B.C., Smith J., Brown T., Taylor R., Hanein Y. (2015). Atomic-Oxygen-Durable and Electrically-Conductive CNT-POSS-Polyimide Flexible Films for Space Applications. ACS Appl. Mater. Interfaces.

[25] Wang X., Li Y., Qian Y., Qi H., Li J., Sun J. (2018). Mechanically Robust Atomic Oxygen-Resistant Coatings Capable of Autonomously Healing Damage in Low Earth Orbit Space Environment. Adv. Mater..

[26] Xu H., Cao X., Shi Y., Cong T., Liu H., Gao Y. (2023). In situ formation of POSS layer on the surface of polyimide film and anti-atomic oxygen of SiO_2_/POSS coatings. Prog. Org. Coat..

[27] Minton T.K., Schwartzentruber T.E., Xu C. (2021). On the Utility of Coated POSS-Polyimides for Vehicles in Very Low Earth Orbit. ACS Appl. Mater. Interfaces.

[28] Gong Y., Tian H., Niu B., Xing Y., Liang X., Zhang Y., Long D. (2024). Molecular Design of Polyimide Films for Combating Atomic Oxygen Erosion Through Combining Experiments with Simulations: A State-of-the-Art Review. Polym. Degrad. Stab..

[29] Chen S., Wu X., Zhong M., Yan D., Huang W. (2023). The atomic oxygen resistant study of a transparent polyimide film containing phosphorus and fluorine. Appl. Surf. Sci..

[30] Zhang Y., Liao B. (2023). Atomic oxygen degradation of a fluorine-containing colorless polyimide. Polym. Degrad. Stab..

[31] Dworak D.P., Banks B.A., Karniotis C.A., Soucek M.D. (2006). Evaluation of Protective Silicone/Siloxane Coatings in Simulated Low-Earth-Orbit Environment. J. Spacecr. Rocket..

[32] Cooper R., Upadhyaya H.P., Minton T.K., Berman M.R., Du X., George S.M. (2007). Protection of polymer from atomic-oxygen erosion using Al_2_O_3_ atomic layer deposition coatings. Thin Solid Films.

[33] Wang Z., Ren X., Zhang Y., Yang C., Han S., Qi Y., Liu J. (2024). Preparation and Properties of Atomic-Oxygen Resistant Polyimide Films Based on Multi-Ring Fluoro-Containing Dianhydride and Phosphorus-Containing Diamine. Polymers.

[34] Song G., Li X., Jiang Q., Mu J., Jiang Z. (2015). A Novel Structural Polyimide Material with Synergistic Phosphorus and POSS for Atomic Oxygen Resistance. RSC Adv..

[35] Banks B.A., Backus J.A., Manno M.V., Waters D.L., Cameron K.C., de Groh K.K. (2011). Prediction of Atomic Oxygen Erosion Yield for Spacecraft Polymers. J. Spacecr. Rocket..

[36] Gotlib-Vainstein K., Gouzman I., Girshevitz O., Bolker A., Atar N., Grossman E., Sukenik C.N. (2015). Liquid Phase Deposition of a Space-Durable, Antistatic SnO_2_ Coating on Kapton. ACS Appl. Mater. Interfaces.

[37] Rahmani F., Nouranian S., Li X., Al-Ostaz A. (2017). Reactive Molecular Simulation of the Damage Mitigation Efficacy of POSS-, Graphene-, and Carbon Nanotube-Loaded Polyimide Coatings Exposed to Atomic Oxygen Bombardment. ACS Appl. Mater. Interfaces.

[38] Zeng F., Peng C., Liu Y., Qu J. (2015). Reactive Molecular Dynamics Simulations on the Disintegration of PVDF, FP-POSS, and Their Composite during Atomic Oxygen Impact. J. Phys. Chem. A.

[39] Tersoff J. (1988). Empirical interatomic potential for silicon with improved elastic properties. Phys. Rev. B.

[40] Brenner D.W. (1990). Empirical potential for hydrocarbons for use in simulating the chemical vapor deposition of diamond films. Phys. Rev. B.

[41] Rahnamoun A., van Duin A.C.T. (2014). Reactive molecular dynamics simulation on the disintegration of Kapton, POSS polyimide, amorphous silica, and teflon during atomic oxygen impact using the ReaxFF reactive force-field method. J. Phys. Chem. A.

[42] Gouzman I., Grossman E., Verker R., Atar N., Bolker A., Eliaz N. (2019). Advances in Polyimide-Based Materials for Space Applications. Adv. Mater..

[43] Liu Y., Li G. (2010). Numerical simulation on atomic oxygen undercutting of Kapton film in low earth orbit. Acta Astronaut..

[44] Banks A.B., Groh D.K.K., Miller K.S. (2004). Low Earth Orbital Atomic Oxygen Interactions with Spacecraft Materials. MRS Proc..

